# Taking working memory training from the laboratory into schools

**DOI:** 10.1080/01443410.2013.797338

**Published:** 2013-05-10

**Authors:** Joni Holmes, Susan Elizabeth Gathercole

**Affiliations:** MRC, Cognition and Brain Sciences Unit, Cambridge, UK

**Keywords:** academic performance, intervention, working memory, memory, primary

## Abstract

Working memory skills have been shown to be enhanced by adaptive training in several randomised controlled trials. Here, two field trials were conducted in which teachers administered working memory training to their own pupils in school. Twenty-two children aged 8–9 years participated in Trial 1. In Trial 2, 50 children aged 9–11 years with the lowest academic performance completed training. They were matched with a group of 50 children who were not trained. Following training, children in Trial 1 improved significantly in both trained and untrained working memory tasks, with effect sizes comparable to those reported in research studies. Improvements on the trained tasks in Trial 2 were comparable, and training was associated with significantly greater progress at school across the academic year in maths and English. These findings indicate that teacher-administered training leads to generalised and robust gains in working memory and educationally significant gains in academic performance.

## Introduction

In recent years, researchers have been developing ways to remediate the cognitive difficulties associated with poor educational progress, with programmes such as Fast ForWord (Tallal, Merzenich, Miller, & Jenkins, 1998), Tools of the Mind (Diamond, Barnett, Thomas, & Munro, 2007) and phonological awareness training (Torgeson, Morgan, & Davis, 1992). One approach that has received particular attention over the past decade is intensive training that focuses on specific core cognitive skills, sometimes termed ‘brain-training’. Regular and prolonged practice has been shown to lead to enduring changes in cognition which reflect plasticity in relevant brain networks. With positive training effects already reported for the ageing population (Buschkeuhl et al., 2008), individuals with anxiety disorders (Schmidt, Richey, Buckner & Timpano, 2009) and children and adults with disorders of memory and attention (Klingberg, 2010), this approach holds the promise of providing a cost-effective method for remediating the cognitive deficits associated with poor educational progress.

The focus of the present research is on the effects of cognitive training on working memory, a cognitive system that appears to play a vital role in academic learning (e.g. Gathercole, Brown, & Pickering, 2003; Geary, Hoard, Byrd-Craven, & De Soto, 2004; Swanson & Siegel, 2001). It provides the temporary storage of information necessary for ongoing complex cognitive activities (e.g. Baddeley, 2000), and is highly associated with measures of academic performance (e.g. Gathercole, Pickering, Knight, & Stegmann, 2004; Jarvis & Gathercole, 2003). Working memory is taxed by many classroom activities such as following instructions, performing tasks that require combining cognitive processing with storage, and seeing complex tasks through to completion (e.g. Gathercole & Alloway, 2008; Gathercole et al., 2008; Gathercole, Lamont, & Alloway, 2006).

Poor working memory has measurable impacts on educationally relevant measures of children's performance. It is a common feature of educational underachievement (e.g. Gathercole et al., 2004) and a substantial majority of children with poor working memory skills fail to meet expected standards in either reading or maths or, most commonly, both (Gathercole & Alloway, 2008). Children recognised by their schools as having Special Educational Needs (SENs) are six times more likely to have working memory impairments than children without SEN (Pickering & Gathercole, 2004). Poor working memory therefore appears to place a child at high risk of poor scholastic attainment.

Training programmes that directly target working memory provide important evidence that it is possible to make enduring changes to these memory abilities. Cogmed Working Memory Training (CWMT) provides intensive practice on a range of computer-based memory tasks for 20–25 sessions (Klingberg, Forssberg, & Westerberg, 2002; Klingberg et al., 2005). The difficulty level of each task is adjusted on each trial to ensure that the individual is working at his or her personal limits. Improvements in working memory following CWMT have now been reported for a variety of populations including typically developing pre-school- and primary-school-aged children (Holmes, Dunning, Gathercole, 2012a; Thorell, Lindqvist, Bergman, Bohlin, & Klingberg, 2009), children with Attention Deficit Hyperactivity Disorder (ADHD) who display elevated levels of hyperactive and inattentive behaviours (Beck, Hanson, Puffenberger, Benninger, & Benninger, 2010; Holmes et al., 2010; Klingberg et al., 2005), children with cochlear implants (Kronenberger, Pisoni, Henning, Colson, & Hazzard, 2011), adolescents with extremely low birth weight (Løhaugen et al., 2011), healthy young adults (Holmes, Dunning, Gathercole, 2012b) and adults with acquired brain injury (Johansson & Tornmalm, 2012; Lundqvist, Grundström, Samuelsson, & Rönnberg, 2010; Westerberg et al., 2007).

In children with poor working memory, Cogmed training boosts memory performance well into the typical-for-age range for the majority of children, and the gains persist for at least six months after training ceases (Dunning, Gathercole, & Holmes, 2012; Holmes, Gathercole, & Dunning, 2009). There is also preliminary evidence of accelerated learning following training, with significant improvements in maths scores reported several months after training for children with working memory impairments (Holmes et al., 2009) and improvements in reading comprehension reported post-training for children with SEN (Dahlin, 2010).

Although these findings suggest that memory gains may benefit the ability to learn, they have so far been demonstrated only in tightly controlled research studies in which the training is implemented by experienced researchers under optimal and often resource-intensive conditions that cannot feasibly be achieved in non-research usage. These trials do not provide evidence for the benefits of training under the real conditions in which it will be used. It is vital now to establish whether the approach can be extended to children who are at high educational risk, or to whole classes, without additionally resourced specialised support. Here, we report findings from two field trials in which teachers administered training to their own pupils. In Trial 1, a whole class of children aged 8–9 years received training and their performance on a range of working memory tasks was assessed before and after training. Previous evidence shows that researcher-led training leads to improvements in non-trained tests of visuo-spatial short-term memory and verbal and visuo-spatial working memory (e.g. Holmes et al., 2009). The aim of this trial was to assess whether the same pattern of generalisation to untrained memory tasks occurs when training is implemented by a teacher. In Trial 2, the impact of teacher-led training on end-of-year school assessments was evaluated with children with poor academic performance. The aim of this trial was to investigate whether school-led training was associated with improvements in academic abilities. Nationally recognised achievement tests, which are used to monitor ongoing school progress and identify children at risk of underachievement, were used to provide educationally relevant measures of performance.

## Trial 1

### Method

#### Participants

All 22 children in a mixed-ability Year-4 class (mean age 8 years 8 months, SD = 4.12, 10 boys) attending a primary school in the South of England participated in training. No children were prevented from participating due to visual, motor or hearing problems.

#### Procedure

School computing staff installed the CWMT software and a member of the research team fully qualified in the administration of the programme provided four hours of training to two members of staff. The pupils were trained in a single group of 22 children in the school IT suite at the beginning of each school day, and supervised by their class teacher and a classroom assistant. Participating children were assessed on an individual basis on eight standardised working memory tasks by a member of the research team, both before and after the training. The researcher conducting these assessments was blind to the intervention. Two children were absent for the post-training tests. Ethical approval was obtained through the University of York Psychology Ethics Committee and consent for participation was obtained from parents/guardians, children and school staff prior to the trial commencing.

*Materials. CWMT.* The RM version of CWMT, published by Pearson Education, was employed (see http://www.psychcorp.co.uk/Education/BestsellingInterventions/CogmedSchools/CogmedWorkingMemoryTrainingSchools.aspx). The standard protocol, in which children complete 20–25 training sessions, was adopted. In each session, children trained on eight different computer-based working memory tasks, completing a total of 120 trials per session. The difficulty of the training tasks adapted to match the child's current ability on a trial-by-trial basis. The programme included a number of motivational and reward features to increase compliance, which included frequent positive verbal feedback, a high score list for each task and a racing game which the children played on at the end of each training session. Training performance was uploaded to a secure server on every day of use and was accessible to school staff for the purposes of monitoring children's progress on the programme. Staff were encouraged to support each participant during the training period through feedback and encouragement, and to provide rewards such as extra play time, stationery items or access to a particular toy or game during free time, for every five training sessions completed (approximately once a week).

*Automated Working Memory Assessment (AWMA).* The AWMA (Alloway, 2007) provided multiple standardised tests of four aspects of working memory. Two tests of each aspect of working memory were used pre- and post-training: verbal short-term memory (Digit recall and Word recall), visuo-spatial short-term memory (Dot matrix and Block recall), verbal working memory (Backward Digit recall and Counting recall) and visuo-spatial working memory (Mr. X and Spatial recall).

### Results and discussion

#### Compliance

Table 1 shows the number of children completing at least 20 training sessions, which is the minimum number required by the Cogmed protocol. It also summarises the Cogmed Improvement Index (CI) for the sample. This is calculated by subtracting the Start Index (average performance across the second and third training sessions) from the Maximum Index (average performance on the two best training days) on two of the training tasks.

**Table 1.** Percentage of children who completed 20+ training sessions and gains on the training tasks, as a function of group.

Trial	% completed 20+ sessions	Cogmed improvement index: all trainees	Cogmed improvement index: completed 20+ session
1	91	21.95 (8.71)	22.72 (7.66)
2, Year 5	80	22.56 (7.31)	21.70 (6.38)
2, Year 6	80	25.60 (8.45)	25.60 (8.69)

Over 90% of the participants successfully completed the standard training protocol; 80% typically complete this in controlled experimental trials (e.g. Holmes et al., 2009). A one sample t-test revealed that the average CI score for children in this trial was not significantly different to that reported in research trials; >.05 when compared to the average CI of 24 (Bennett, Holmes, & Buckley, 2013; Holmes et al., 2009, 2010). CI scores did not differ significantly for children completing ≥ 20 or <20 training sessions, *t*(18) = 1.230 and *p* = .245. Additionally, there was no significant correlation between the number of sessions completed and the gains on the training tasks as measured by the CI (*p* >.05). On this basis, all children were included in subsequent analyses irrespective of number of training sessions completed.

**Figure 1.**
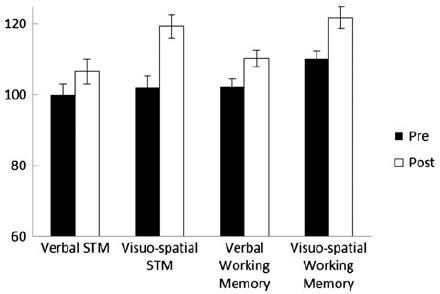
Mean pre- and post-training working memory scores for Trial 1.

Figure 1 shows the mean pre- and post-training scores across the two subtests for each of the four aspects of working memory. There were significant gains across all four aspects of working memory, with Cohen's *d* effect sizes of .43, 1.12, .75 and .94 for verbal short-term memory, visuo-spatial short-term memory and verbal and visuo-spatial working memory, respectively (*ps* from .029 to .001). Scores on individual subtests before and after training are displayed in Table 2. There were significant gains across all measures except Digit recall and Counting recall. On average, children moved from the 63rd to the 87th centile following training.

**Table 2.** Impact of training on individual working memory subtests in Trial 1.

	Pre		Post				
	*M*	SD	*M*	SD	*t*	*p*	*d*
Digit recall	100.35	16.73	106.25	17.62	1.91	0.07	0.34
Word recall	100.75	17.00	106.85	15.56	2.12	0.05	0.37
Dot matrix	102.90	20.65	122.30	18.18	4.24	0.00	1.00
Block recall	103.30	16.87	116.20	13.64	2.77	0.01	0.85
Backward digit recall	100.50	10.34	116.30	13.24	4.40	0.00	1.34
Counting recall	104.85	13.90	104.15	12.76	0.19	0.85	0.05
Mr. X	109.65	14.08	122.90	19.99	2.82	0.01	0.78
Spatial recall	108.40	13.77	120.70	11.44	2.93	0.01	0.98

One key issue is the extent to which the magnitude of training gains varied as a function of working memory skills at baseline. To explore this, the participants were first split into two groups on the basis of their baseline working memory scores. Baseline scores were calculated by taking the mean of the pre-training scores for the four aspects of working memory for each participant. The sample was then split into two subgroups around the median of 106: low baseline *M* = 97.013, SD = 7.258 and high baseline *M* = 113.955, SD = 5.034. A MANO VA conducted on gain scores in each of four areas of working memory revealed a significant group effect, *F*(4, 14) = 4.695, *p* = .013 and *η* = .573. Univariate analyses established that children with low baseline scores made greater gains than the high baseline group following training on measures of visuo-spatial short-term memory, *F*(1, 17) = 6.532, *p* .020 and *η* = .278, and verbal working memory, *F*(1, 17) = 18.064, *p* .001 and *η* = .515.

The school-led training in this trial was therefore associated with improvements in working memory tasks that were independent of the training activities, and training gains were most substantial for children with low working memory skills. The greatest improvements were for visuo-spatial short-term memory and verbal and visuo-spatial working memory, aspects of working memory that are strongly associated with learning (e.g. Gathercole et al., 2004). This raises the possibility that training-related improvements in memory could benefit children's academic progress. The aim of the second trial was to explore this possibility by assessing whether teacher-administered memory training improved school performance in low ability children.

## Trial 2

### Method

#### Participants

Fifty children aged 9–11 years with low academic performance were selected to participate in training. They were selected from a total cohort of 256 Year 5 and Year 6 children attending a middle school in South East England. Selection was based on an average raw score in English and maths from Teacher Assessments administered at the end of the previous school year. These assessments, which are administered annually at the school, are combined with teacher observations to inform judgements about a child's progress measured against Assessing Pupils’ Progress grids. These grids form part of The National Strategies defined by the UK's Department for Education and allow teachers to judge a child's performance against a set of pre-defined criteria. Tracking pupil progress in this way is voluntary in the UK and the Government believes that schools are best placed to decide what assessments to use; there no prescribed approaches to assessment. The school involved in this trial used test questions taken from previous national Standard Assessment Test (SAT) papers, which assess the different areas of English and maths set out in the UK National Curriculum. The English assessment tested reading, writing, speaking and listening skills. The maths assessed a child's ability to use and apply maths, and complete tests of number and algebra, shape space and measures and handling data. Raw scores range from 9 to 35 for children aged 5–11 years, with lower scores reflecting poorer performance.

Twenty-five children were recruited from Year 5 (mean age 9 years 5 months, SD = 3.37, 16 boys) and 25 from Year 6 (mean age 10 years 6 months, SD = 3.9, 13 boys). These children had the lowest Teacher Assessment scores of their respective cohorts. No children were excluded from the study due to difficulties using a computer mouse effectively, or sight or hearing problems. They were matched with a group of 50 children on gender, age (within 30 days) and performance on Teacher Assessments from the previous cohorts of children in Years 5 and 6. For Year 5, the trainees (mean age 9 years 5 months, SD = 3.37) had an average Teacher Assessment score of 20.2 (SD = 3.03). The comparison group's (mean age 9 years 5 months, SD = 3.36) mean score was 21.13 (SD = 2.96). For Year 6, group means were 21.66 (SD = 5.25) for the trained group (mean age 10 years 6 months and SD = 3.91) and 22.52 (SD = 2.27) for the comparison group (mean age 10 years 6 months, SD = 3.82).

Data for the two year groups are presented separately because the Year 5 and 6 assessment points have distinct status in the UK state education system. SATs in English and maths are compulsory in Year 6, as this is the final year of Key Stage 2 which spans 7–11 years, and these measures are widely used as indicators of school progress and added value. In addition, schools can choose to assess pupils at the end of Year 5 using optional SATs, as this participating school did.

#### Procedure

A member of the research team provided four hours training on the use of CMWT to two members of staff. The software was installed by school computing staff. Participants from Year 5 were trained in a single group of 25 in the school computer suite, supervised by the Head teacher and a classroom assistant at the end of the school day. The Year-6 children were trained in two smaller groups (*n* = 12, *n* = 13 respectively), supervised by the same school staff at the end of the school day. Ethical approval for this trial was obtained through the University of York Psychology Ethics Committee and consent for participation was obtained from parents/guardians, children and school staff.

#### Materials

*CWMT.* The RM version of CWMT, published by Pearson Education, was used (see Trial 1 for details).

*Academic outcomes.* For Trial 2, progress in English and maths during the year of intervention was measured by performance against National standards in both areas, which are defined by the Department for Education's National Curriculum levels. These range from 1 to 10 for children in compulsory education in the UK (aged 5–16 years). There are three sublevels within each level (a, b and c). An ‘a’ indicates that a child is performing consistently at a level and is ready to progress to the next, a ‘b’ means that they are secure at a particular level and a ‘c’ means that they are just starting on a level (see http://www.education.gov.uk/schools/teachingandlearning/curriculum/primary). Children are expected to progress by two sublevels per school year and achieve a level 4c or above by the end of Year 6 (age 11). The school participating in Trial 2 provided attainment levels for all children, including those in the comparison groups, both at the beginning and end of the school year. These levels were based on the children's performance on optional SATs in each area. Average sublevel improvements were calculated for all children for the relevant academic year. For example, a child who started the year at 3b and finished at 4a would have an improvement score of four sublevels (3a, 4c, 4b and 4a). The children's classroom teachers, who conducted the end of year assessments, were not aware which children were receiving memory training.

### Results and discussion

#### Compliance

Training compliance and a measure of improvement on the training tasks are shown for all groups in Table 1. As in Trial 1, the majority of children successfully completed the full 20 days training. Average CI scores for children in Years 5 and 6 did not differ significantly to those reported in research trials; all *ps* > .05 when compared to the average CI of 24 (Bennett et al., 2013; Holmes et al., 2009, 2010; Tan-nock et al., 2012). Training performance was unaffected by the number of training sessions completed. CI scores did not differ significantly between children completing >20 or <20 training sessions for those in Year 5, *t*(23) = 1.187 and *p* = .248, or Year 6, *t*(23) = .0 and *p* = 1.0, and there were no significant correlations between the number of sessions completed and the gains on the training tasks for either year group (all *ps* >.05). There were no significant differences in baseline academic performance between children completing <20 and >20 sessions for children in Year 5, *t*(23) = −.887, *p* = .387 and *d* = .6, or children in Year 6, *t*(23) = −1.160, *p* = .259 and *d* = .545. All children were included in subsequent analyses.

Table 3 summarises the gains in attainment sublevels across the relevant academic years (prior to training and following training) for children who received training and the comparison groups who did not receive training. Children in Year 5 who completed training made significantly greater gains in maths than the comparison group, *F*(48) = 14.44 and *p*<.001, but no significant group differences in gains were found in English *F*(1, 48) = 3.93 and *p* = .053.

**Table 3.** Mean sublevel gains (SDs) in attainment as a function subject and school year for Trial 2.

	Year 5			Year 6		
	Trained group	Comparison group	*d*	Trained group	Comparison group	*d*
English	1.48 (1.56)	2.36(1.58)	0.56	2.00 (1.44)	1.12 (1.20)	0.67
Maths	1.36 (1.29)	−1.04 (2.88)	1.15	2.12 (1.13)	1.32 (1.55)	0.60

Children in Year 6 who received training made significantly greater progress both in English, *F*(1, 48) = 5.49 and *p* = .023 and in maths, *F*(1, 48) = 4.36 and *p* = .042. Of the trained group, 84% reached nationally expected levels of attainment (4c and above) in English at the end of Year 6, compared with 72% of the comparison group. These results indicate that school-led memory training can benefit educationally relevant measures of school performance.

To investigate whether progress in academic attainment was related to baseline attainment, children in the trained groups were divided into low and high baseline groups. A median split approach was used. Children in Year 5 with baseline scores <20 and children in Year 6 with scores <21 were classified as having low baseline attainment scores. Children with scores >20.01 in Year 5 and >21.01 in Year 6 formed high baseline groups. MANOVAs with baseline group and academic progress in English and maths entered revealed no significant differences between the groups for children in Year 5, *F*(2, 22) = 2.199, *p* = .135 and *η* = .166, nor for children in Year 6, *F*(2, 22) = .327, *p* = .725 and *η* = .029. Thus, in this trial, the impact of training on academic progress was not mediated by the children's baseline academic performance.

## General discussion

The aim of this study was to investigate whether an intensive computerised programme found to be effective in enhancing working memory performance in researcher-led studies improves memory and learning when implemented in schools by teachers. The results were encouraging. Compliance rates were high, with more than 80% of children across both trials completing 20 sessions of approximately 45 minutes of the programme, a completion rate comparable to previous research studies (e.g. Holmes et al., 2009). Crucially, improvements on the training activities were equivalent to those observed in research trials, both for a mixed-ability class of children and for low-achieving children who were trained in group sizes ranging from 12 to 25. It is therefore feasible for working memory training to be conducted with reasonably large groups of children in school, with both high rates of compliance and remarkably good rates of progress on trained activities.

The training gains for the whole-class trial of children aged 8–9 years extended across a range of untrained standardised measures of working memory. Significant improvements were found in all assessed aspects of working memory, but were greatest for tasks that required children to recall sequences of visuo-spatial information or simultaneously hold in mind and manipulate sequences of verbal or visuospatial information. These tasks, which closely resemble the trained tasks, are strongly associated with the ability control and focus attention in cognitively demanding situations (e.g. Kane & Engle, 2003). They are also highly predictive of children's learning abilities across the school years (Gathercole et al., 2004; Jarvis & Gathercole, 2003). It is therefore possible to modify these important, basic working memory abilities through group-based training with a teacher in school.

There was evidence from the second trial with low-achieving children aged from 9 to 11 years that school-led memory training enhances children's academic performance. Trained Year 6 children made significantly greater progress across the academic year in English (speaking, listening, reading and writing skills) than matched untrained pupils, and a greater proportion of the trained group reached target levels of attainment in National Curriculum tests in this area at the end of the school year. Training was also associated with greater advances in maths attainment levels across the year of the intervention for low-achieving children in both Year 5 and Year 6. It should, however, be noted that for the younger children, this reflected a drop in performance across the school year for the comparison group.

Finding that training gains transfer to improvements in National Curriculum assessments in English and maths provides a crucial step in the consideration of cognitive training as an educational intervention. Many studies report that extensive training on highly artificial working memory tasks benefits performance on other rarefied memory tasks administered under controlled conditions (see Klingberg, 2010 for a review), but it has been difficult to demonstrate that these gains transfer to meaningful improvements in other skills and abilities (see Holmes, 2011, and Shipstead, Redick, & Engle, 2010 for reviews). The present results establish that memory training has the potential to transfer to educationally relevant measures of academic ability, even when conducted under real-life conditions in schools. Because working memory difficulties are increasingly recognised as a hallmark feature of specific learning difficulties (e.g. Rose, 2009), as well as slow rates of learning more generally (e.g. Gathercole & Alloway, 2008), these findings have practical implications. The educational gains and cost-savings of using memory training as an early intervention could be immense and these promising results certainly warrant further exploration. A priority for future studies is to establish whether these gains transfer to larger-scale, possibly whole-school, interventions with controlled randomised trials methodology to establish more precisely the value of school-implemented working memory training.
